# Evaluation of a car-based method for detecting the invasive *Lupinus polyphyllus* Lindl in road infrastructure

**DOI:** 10.1038/s41598-026-58979-w

**Published:** 2026-06-25

**Authors:** Juliana Dániel-Ferreira, Jan Olof Helldin, Tommy Lennartsson

**Affiliations:** Swedish University of Agricultural Sciences, Swedish Biodiversity Centre, Box 7012, Uppsala, SE-75007 Sweden

**Keywords:** Invasive alien plants, Detection probability, Road verge management, Environmental monitoring, Ecology, Ecology, Plant sciences

## Abstract

Growing recognition of the negative impacts of invasive alien plants on native species diversity and ecosystem function has led to greater focus on effective management strategies. Monitoring is crucial for both prevention of establishment and planning of eradication of established IAPs. However, monitoring is often constrained by resource limitations, and less time-consuming methods risk providing too low levels of detection. *Lupinus polyphyllus* is a highly problematic non-native plant species in Europe. We evaluated the detection performance of a car-based survey method for finding *L. polyphyllus* in road verges and how the physical characteristics of the stands influenced their detection from the car. At the 200 m scale, 85% of sections containing *L. polyphyllus* were correctly identified, while 72% of individual stands were detected. Results indicated that detection probability was influenced by stand density and position in the road verge. Dense stands had the highest detection probability, while single plants were less likely to be detected. Stands present in side and back slopes were more likely to be detected than those only in one slope, with the side slope stands having the lowest detection probability. Our study shows that the car-based survey is a reliable large-scale method for detecting *L. polyphyllus* and for providing a general estimate of its abundance in road verges. Such information is useful for designing management strategies to hinder seed production and dispersal of the invasive. Since the detection success of single plants was lower, more detailed surveys at local scales are recommended for early eradication.

**Supplementary Information:**

The online version contains supplementary material available at 10.1038/s41598-026-58979-w.

## Introduction

Invasive alien species (IAS) are species that have been introduced beyond their native range and have become problematic in the novel ecosystems due to rapid expansion, often linked to lack of natural enemies. IAS therefore pose a threat to ecosystems worldwide^1,2^. Among them, invasive alien plants (IAPs) have been shown to reduce native species abundance and diversity, and can even alter ecosystem function^3^. Recent projections indicate that the rate of new introductions is rising, underlining the urgency of effective management strategies to mitigate this global challenge^4^. This exerts a growing pressure on governmental agencies and environmental managers to tackle a wide array of challenges that emerge at the different stages of the invasion process^5^. Although prevention (i.e. early detection, rapid response, and eradication) is the main strategy for control of IAS^6,7^, other management strategies need to be applied for the control of species that have already established and are at the late stages of the invasion process^7^. At these stages, monitoring the current state of the invasion and the efficacy of management measures, as well as anticipating where the species will spread becomes increasingly important^6,8^. Monitoring is also essential for keeping track of potential IAP species.

Extensive monitoring requires considerable resources, and financial constraints can hinder comprehensive monitoring efforts, potentially leading to gaps in data collection^9^. Furthermore, probability of detection during field surveys is often imperfect and varies depending on several factors. For plants, detection variability during surveys could originate from the observers and their level of experience^10^, size and growth form of the stand^11^, conditions specific to the type of survey and environment^11^, and interactions among these factors^12^. Despite these challenges, effective long-term monitoring of IAP remains essential for informed decisions in ecosystem management^13^. Failure to detect IAP results in the spread of the species and increases costs because implemented management strategies risk to miss their targets^12^. Therefore, it is important to explore survey methods to establish their cost-effectiveness and the variation in the probability of detecting the target species.

Road verges have been shown to be an important conduit for the expansion of IAP^14–16^. Seeds can disperse over long distances along the road network through the movement of vehicles^17^ and through road building and maintenance^18^. To monitor the status and spread of IAP along road verges, researchers and practitioners have employed different approaches such as remote sensing using satellite e.g. Lourenço^19^, or aerial^20^ imagery, georeferenced imagery, e.g. Google Street View^21^, as well as regular field surveys e.g., Joly^22^. Vehicle-based surveys conducted from moving vehicles have also been used to detect other IAP along road networks in other regions (e.g.^23,24)^. While each method provides distinct advantages over the others, they often also have limitations and are thus not appropriate in all situations. For instance, remote sensing images are easy to access and have broad spatial coverage, but the images are often too coarse and their analysis can be challenging due to similarity among species^25^. Furthermore, detection of certain plant species is generally facilitated by conducting the survey at the right time, preferably when the plants are distinguishable through phenologically prominent features such as flowering stage^13^. While this applies to most vegetation survey methods, approaches that rely on imagery collected for other purposes are often constrained by fixed acquisition dates. In contrast, field-based surveys can be intentionally timed to coincide with peak detectability.

The Swedish Transport Administration (STA) routinely surveys selected road verges for IAP. The goal of the surveys is to detect new occurrences for eradication and also to monitor the spread and distribution of the species in the road network. However, the survey method varies among regions in the country. Here, we present and evaluate a car-based survey method intended for use within road management organisations that already use moving vehicles to monitor roads and road verge vegetation. Consistent use of a single survey method could facilitate comparability of data among regions and enable a more seamless collaboration between practitioners and researchers. The evaluation is based on a comparison with more detailed surveys conducted on foot, which are used here as a reference method rather than as a comprehensive inventory approach.

*Lupinus polyphyllus* (hereafter lupine) is one of the most problematic non-native species in Europe due to its environmental and economic impacts^26^. Despite it not being included in the European Union’s list of invasive alien species of conservation concern, the species is considered invasive and the Swedish Environmental Protection Agency is striving to reduce its spread^27^^,28^. Given that lupine is the most common IAP in the Swedish road network, we developed a method to detect the species along road infrastructure. Since the method is designed to be used to survey the entire road network in a region, it needs to be fairly fast without substantially reducing detection performance. Therefore, a car-based method was designed, which both has the potential to fulfil these requirements, and aligns to the routines for road monitoring already in use by the STA. We evaluated the detection performance of the method, as well as which characteristics of the lupine stands can affect their detection probability.

We hypothesize that the factors likely affecting the detection rate of lupines in our survey method are the density of plants in a stand, the location of the stand in the road verge, and whether the stand occurs both in the road verge and outside the road area or not.

## Methods

### Survey method and areas of study

To perform the survey, all accessible road verges within the selected area were scanned for lupines from a car with two people. One person drove the car while the other looked for the invasive along the road verges and registered the surveyed roads and occurrences of the species in a digital map and protocol. The recommended speed ranged between 20 and 50 km/h, depending on the abundance of stands and the width of the road. The speed was reduced as the abundance of lupines in the road verge increased or the visibility decreased, and if necessary the car could stop completely next to a stand (e.g. when the abundance of lupines was too high to allow reliable recording while moving, or to improve positional accuracy in areas with poor signal). If the road was sufficiently narrow to allow good visibility of both road verges, the observer was advised to register the stands present in both sides of the road whilst driving through the road only one time. However, if the road was wide and visibility of the road verge on the other side was impeded, the survey was performed for each side of the road separately. The method is described in detail in Helldin et al.^29^. All surveyors had a background in ecology or biology and received training prior to the start of the survey.

**Fig. 1:**
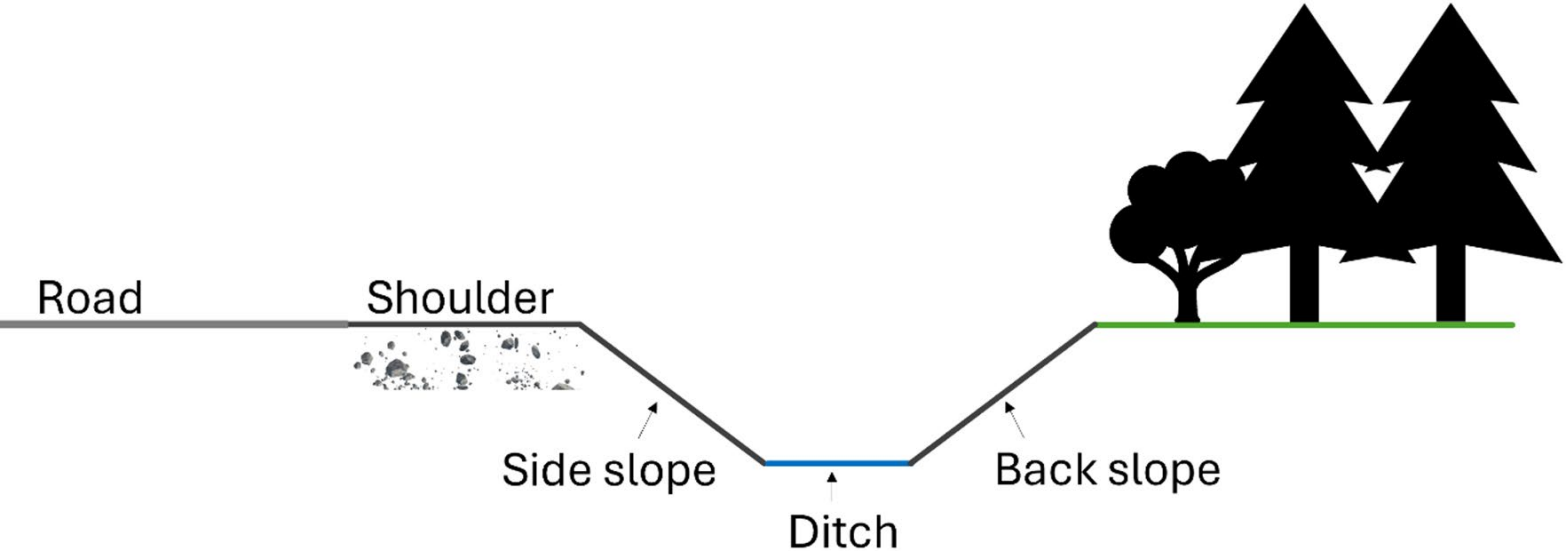
Illustration of a cross section of a road and the road verge. Plants of *L. polyphyllus* could be observed in the side slope, the back slope, or both. Stands could also continue outside of the road verge.

The goal of the survey method was to collect data on both the distribution and the abundance of lupine in the selected regions. The abundance was measured by the total number of occurrences, their size, and also the density of plants within a stand. Using the ESRI app Field Maps (earlier Collector), each stand was marked on a basemap with a polyline object that marked its beginning and end. Through the Field Maps protocol, each line object also contained information about the density, position, and width of the stand. It was also registered whether the stand continued outside of the road verge into the neighbouring habitat or not. The density was selected by the observer as one of the following categories: individual plant(s), sparse stand (the IAP covered less than 50% of the ground surface in the stand in vertical projection), dense stand (the IAP covered between 50 and 90%) and dominating (the IAP covered more than 90%). Stand density can be assumed to be important for the detectability, as well as for the effects of lupine on plant communities^30,31^. Plant density is difficult to assess^32^ and can easily become subjective. Initial tests of the vertical projection method indicated that by thorough examination of each stand, coverage differences of c. 25% could be detected with high reproducibility cf. Kennedy and Addison^33^. However, such examination was too time consuming for a method aiming at rapid surveying of large areas. Instead, we defined broad density categories that can be assessed under field conditions during car-based surveys, while still reflecting major differences in plant cover. Very high coverage proved to be readily discernible irrespective of observer, as well as the boundary above/below 50%; the latter mainly due to the fact that density in those categories in most cases was considerably above or below 50% of cover. The position of the stand could be one of three choices: side slope (i.e. the road embankment), back slope, or both side and back slopes (Fig. 1).

For simplicity, the width of the stand was an approximation given by the observer as an integer starting at 1 m. The width of the stand could subsequently be used to create polygons to acquire a more comprehensive representation of the abundance of the species, as well as an estimate of the area in need of control measures. A new line object was created if there was a break in the occurrences or if there was a pronounced change of stand width. The length of the gap needed to register a new stand depended on the circumstances. With a low number of plants, a 10 m gap was applied. This threshold reflects both GPS uncertainty and seed dispersal in lupines, which can disperse over distances up to 5.5 m from the mother plant^26^. By applying a 10 m threshold, we aimed to avoid observations that represent the same stand and to decrease the overall survey time. If it became too time consuming, the occurrences were registered as a single line object with patchy occurrences (i.e. a yes/no question in the protocol related to the patchiness of the stand). A new line could also be registered if there was a clear change in density. This was only relevant for larger stands with reasonably homogeneous density, for example where there was a large dense core stand that transitioned into sparser or varied stands. In the case of smaller occurrences with different densities (which are < 10 m apart), a line with varied density was registered.

**Fig. 2:**
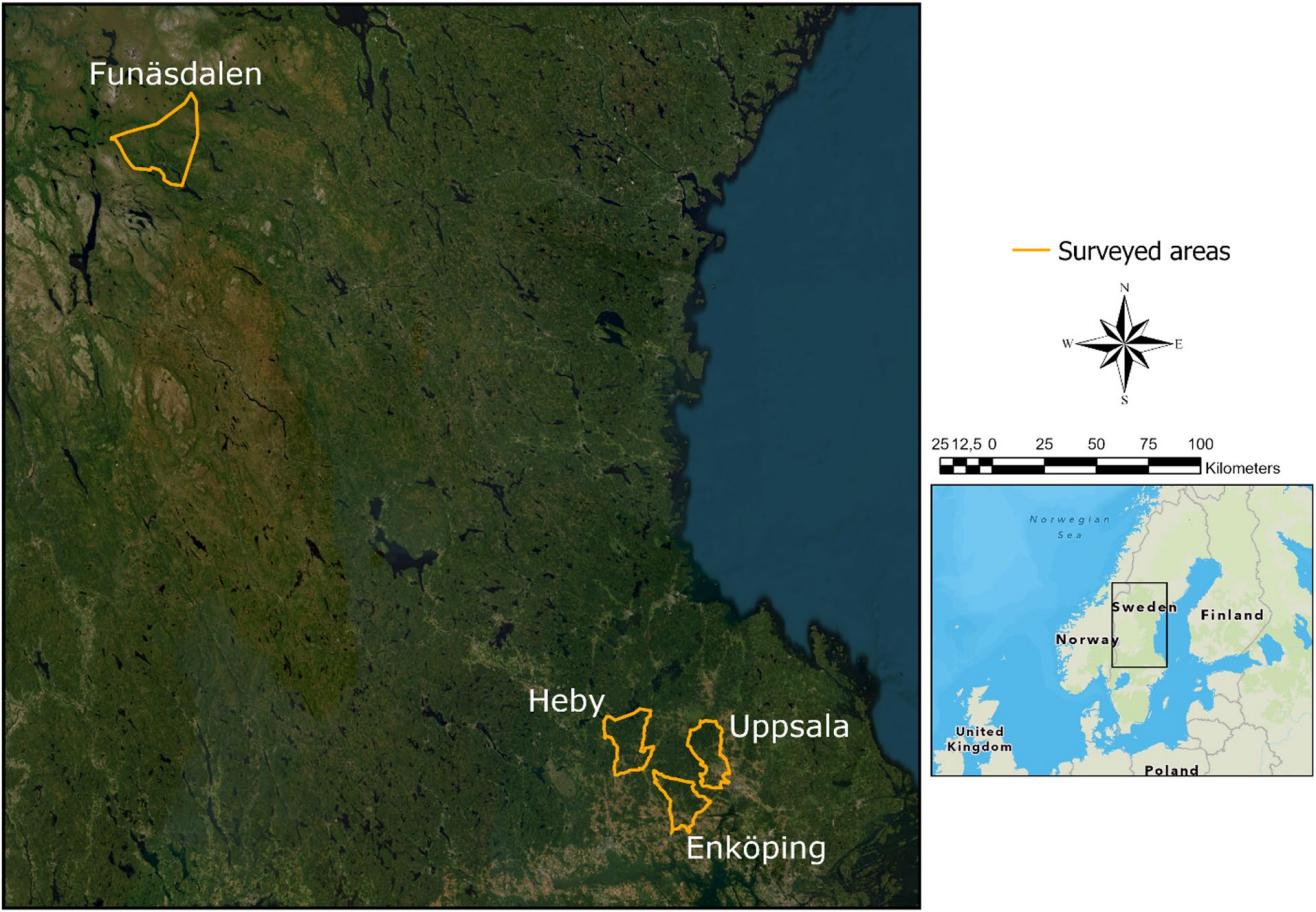
Map of the four areas (Heby, Enköping, Uppsala, and Funäsdalen) surveyed using the survey by car method and verified by the survey by foot method.

The survey was conducted in four areas during the peak season of lupine flowering, which is early June in the southern study areas, and late July in the northern study area (Fig. 2).

All observed occurrences of lupines, both flowering and vegetative individuals, were registered during the survey. Three of the areas were located in the boreo-nemoral lowland in south-central Sweden, to the west of the city of Uppsala, and the fourth was located in a subalpine area further north, around Funäsdalen in the province of Härjedalen. The areas were selected to represent a variation in overall lupine abundance and to include different landscape types, subalpine forest, lowland forest and lowland agricultural. Two of the lowland areas (Heby and Uppsala) were surveyed in 2021. In the summer of 2022, the Heby area was extended, and some areas were surveyed again. The Funäsdalen area was surveyed in 2022. During 2023, the third lowland area, Enköping, was surveyed (Fig. S1–S4). Surveys were timed to coincide with peak flowering times of lupines and conducted prior to roadside mowing to maximise detectability. Accordingly, the three regions in south-central Sweden were surveyed in June, while the region in Funäsdalen, with a later phenological development, was surveyed in July.

### Evaluation of the survey method

To assess the type and proportion of lupine stands that were detected during the survey by car, we selected 131 verification stretches, i.e. shorter road stretches (200 m) where a more detailed survey was conducted by foot instead of by car (~ 26.2 km in total). During these more detailed inspections, the surveyors walked the length of the stretches along both sides of the road at a slow pace while searching for lupines on the road verge from a closer distance and without obstructed view. We selected the verification stretches at random points along road sections distributed over the respective study area (Fig. S1 – S4). Verification stretches were of two types: (i) *non-empty stretches*: where lupines were recorded during the survey by car, and (ii) *empty stretches*: stretches that were considered empty during the survey by car. For the survey by foot, the observers started by parking the car at the predetermined given points and then proceeded to survey the road verges on both sides 100 m in opposite directions from the given point (i.e., 200 m road stretch and 400 m roadside in total). The observers recorded lupine occurrences using the same Field Maps protocol as during the survey by car (see above).

Occurrences (line objects) recorded during the survey by foot were compared with those registered during the survey by car in ArcGIS Pro 3.1. An examiner compared each stand based on its location, extent, and recorded attributes in the two datasets to determine whether it had been missed during the survey by car or not. Stands were classified as ‘detected’ when there was clear correspondence between observations in the two datasets (Fig. S5). First, stands whose line objects overlapped were considered matches. Second, to account for positional uncertainty, objects located within 10 m of each other were also considered matches where no ambiguity existed. Third, in cases where the spatial correspondence was less clear, an additional manual assessment was conducted to match stand characteristics (e.g. density, position within the road verge). This step was sometimes necessary because positional discrepancies between the two surveys were expected due to GPS uncertainty and in differences in survey conditions, thus allowing the examiner to determine whether differences between the surveys reflected true missed detections or variations in how stands were represented in the map.

### Statistical analysis

**Fig. 3:**
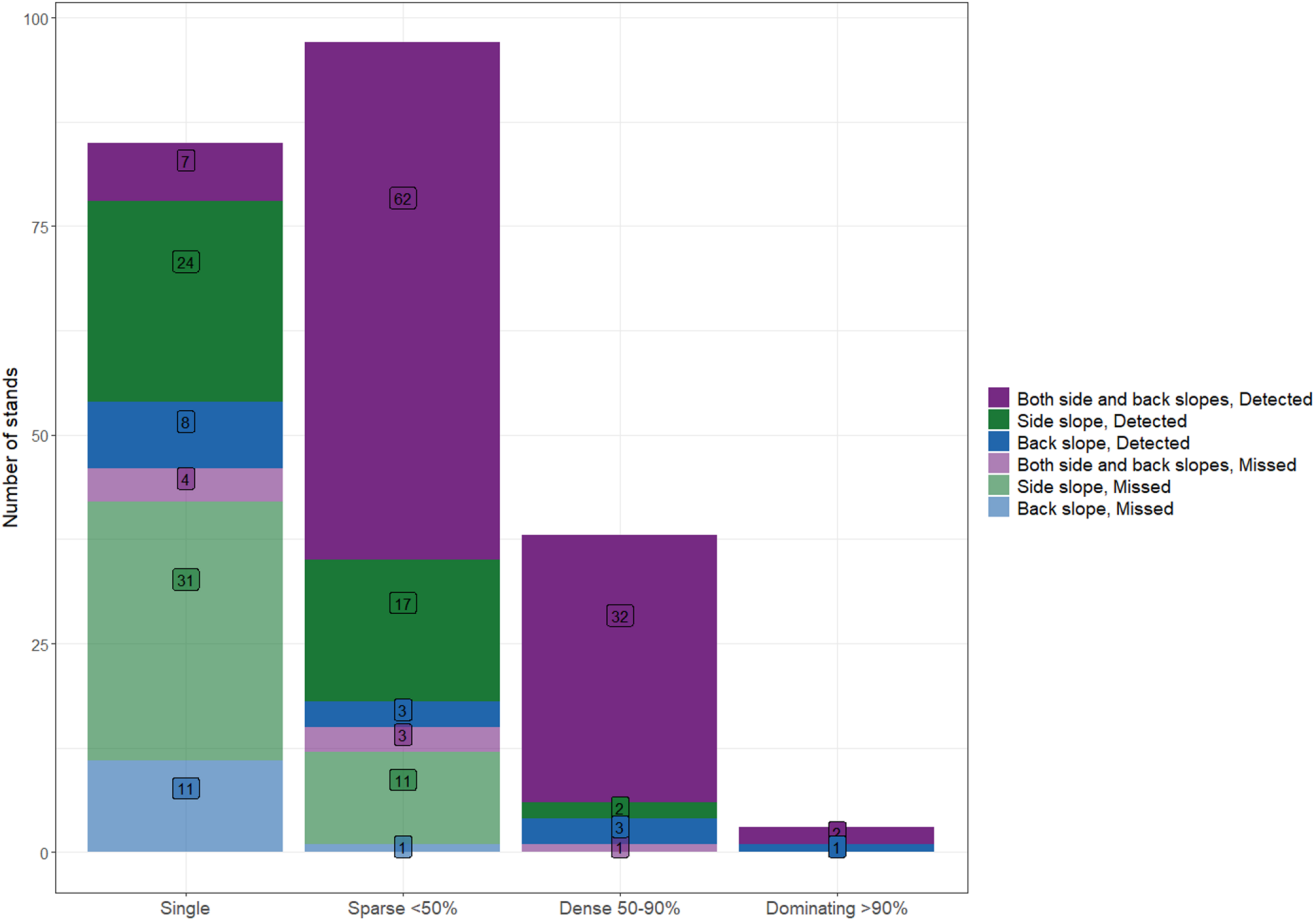
Description of the type of lupine stands, their location in the road verge, and whether they were detected or missed during the survey by car.

Analyses were performed in R 4.1.2 R Core Team^34^. A generalized linear mixed model (GLMM) with a binomial distribution and a logit link was used to investigate which variables influenced the probability of detecting or missing a lupine stand from the car. The response variable was binary: ‘Yes’ was given for a stand that was detected both from the car and by foot, and ‘No’ was indicated for a stand that was detected only during the survey by foot (i.e., the object was not detected during the survey by car). The explanatory variables were the characteristics of the stand as described by the survey by foot: stand location within the road verge (factor), stand density (factor), and whether the stand continued outside of the road verge (factor). In the analysis, the density category ‘dominant’ was removed because there were only three observations along the verification stretches and all of them were detected from the car (Fig. 3). The stand location was of interest because we assumed that plants present in the side slope could be more easily missed when viewed from the road. The density of the stand was of particular interest as we assumed that denser stands (i.e. with more individuals per m^2^) are easier to detect from the car. A factor indicating whether the stand continued outside of the road verge was added to the model given that this could also be an indicator of the size of the stand. The density was assessed for each stand parallel to the road, while the presence of the stand outside the road verge was an indicator of the size perpendicular to the road, which could also influence the visibility from the car. Finally, the study area was added as a random effect to account for the variability emerging from the differences in the distribution of lupines among areas, differences that may arise from the different observers that performed the survey, and from the survey being.

conducted in the three different years. We did not consider any interactions among the variables of interest to be relevant for this analysis. We also assumed weather and observer identity could influence detection probability, as factors such as precipitation may affect visibility from the vehicle. However, these variables could not be examined due to limited variation in the dataset (most of the data was collected in sunny weather). Finally, we tested survey year as a random effect but did not retain it in the final model because it did not improve model fit and its estimated variance was negligible.

After the model was fitted, it was visually validated using the package *DHARMa*^35^. To investigate exactly what characteristics of the stand were related to a lower detection probability during the car inventory, a post-hoc analysis was performed using the *emmeans* package^36^. To adjust for multiple comparisons, the Tukey method for comparing a family of three estimates was applied. Finally, we compared the fitted model to a null model using Akaike’s information criterion corrected for small sample sizes (AICc).

Finally, given that the speed during the survey by car varied according to the density of lupines in the road verges, we also performed a separate analysis to evaluate how speed influenced the detection of lupines from the car. The speed was only recorded during the first two years of the survey along the verification stretches, and therefore a separate analysis that used only the data from 2021 to 2022 was performed. Vehicle speeds along the verification stretches were recorded in 10 km/h intervals and ranged from 20 to 50 km/h. The highest speed occurred only along one stretch and was therefore excluded from the analysis. We evaluated how an interaction between the explanatory variables explained above (i.e. stand location within the road verge, stand density, and whether the stand continued outside of the road verge) influenced whether the stand was detected or missed during the survey by car. We added the interacting effect to each of the explanatory variables in separate models and calculated the AICc to detect the most parsimonious models.

## Results

**Fig. 4:**
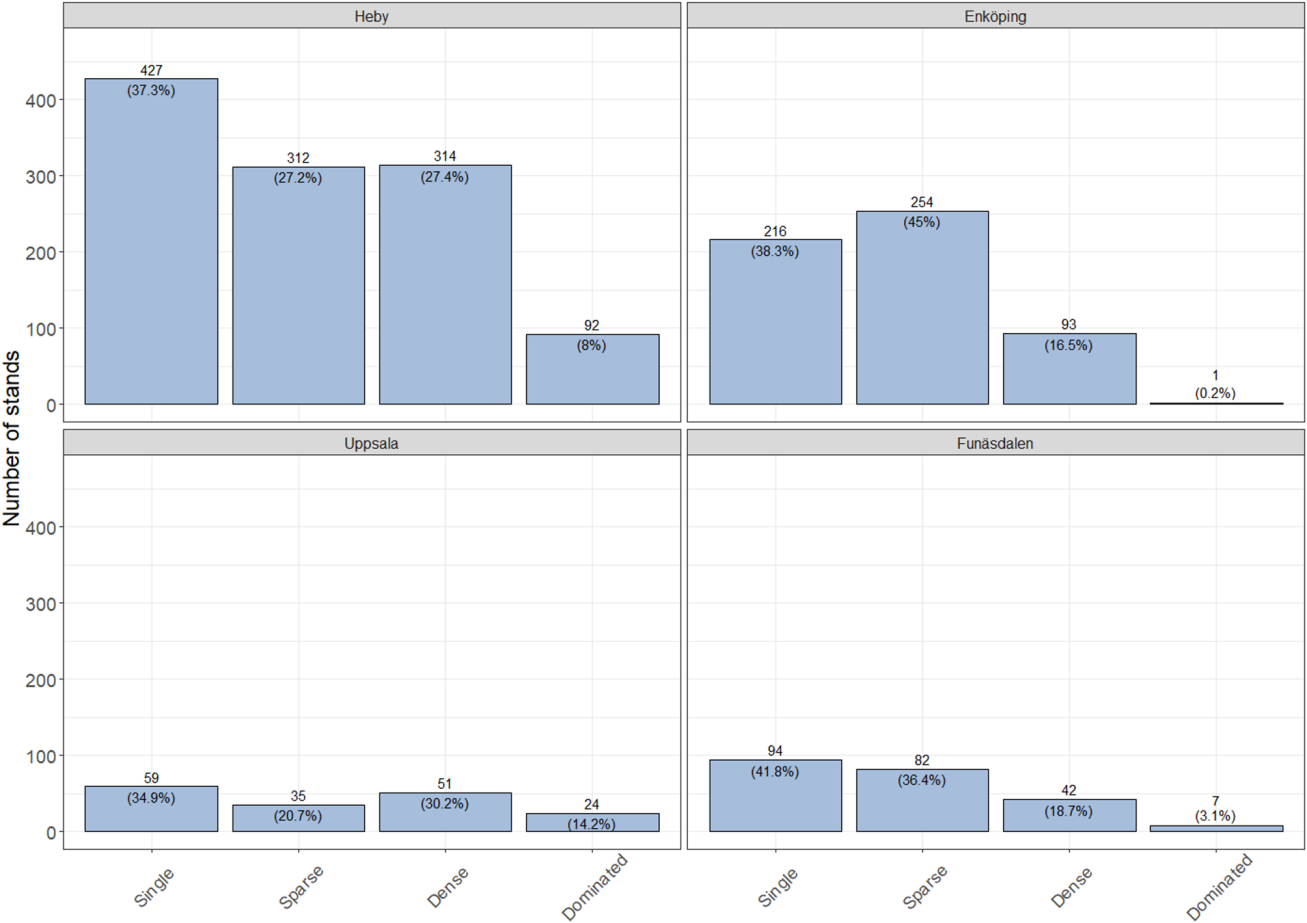
Total number of lupine stands and their plant density in the four surveyed areas in Sweden.

There was a total of 2,103 lupine stands found along 2,933 km within the four areas (Tables 1 and 2; Fig. 4). Of the 131 verification stretches, 61 stretches were identified as empty from the car. Among the 61 stretches classified as empty from the car, lupines were found in nine (14.75%) of these stretches during the survey by foot, meaning that lupines had been missed during the survey by car. Along non-empty stretches, 223 stands of flowering lupine were observed during the survey by foot, and 27.8% (*n* = 62) of them were missed during the survey by car (Fig. 3). In 2021, 46 stands were observed in Uppsala and Heby, and 11 (23.91%) were missed during the survey by car. In 2022, 83 stands were observed in Uppsala, Heby, and Funäsdalen and 36 (43.37%) were missed during the survey by car. In 2023, 94 lupine stands were observed in Enköping and 15 (15.96%) were missed during the survey by car (Table S1). No commission errors were recorded during the survey by car (i.e. no stretches were falsely identified as containing lupines).

**Table 1:** Number of verification stretches (surveyed by foot) per survey area, separated by year and output from the preceded survey by car.

Area	Year of survey	Number of non-empty stretches	Number of empty stretches
Heby	2021	10	10
Heby	2022	20	11
Uppsala	2021	10	10
Uppsala	2022	2	8
Funäsdalen	2022	7	3
Enköping	2023	21	19

**Table 2:** Number of surveyed kilometres per area and year, and the total number of lupine stands registered for each area. Note that the number of lupine stands per kilometre is a rough estimate of the density of the species in the road network within each area.

Area	Km 2021	Km 2022	Km 2023	Total km	Total lupine stands	Lupine stands/km
Heby	667.5	383.9	-	1051.5	1145	1.1
Enköping	-	-	395.4	395.4	564	1.4
Uppsala	723.5	230.6	-	954.1	169	0.2
Funäsdalen	-	532.1	-	532.1	225	0.4

**Fig. 5:**
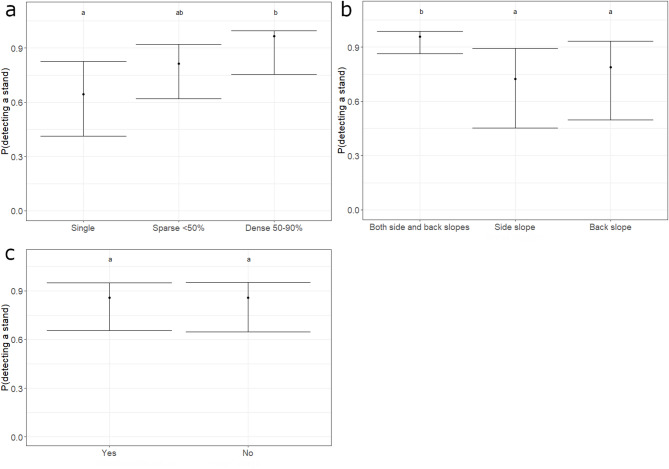
Result of the GLM exploring which characteristics of a lupine stand affect their probability of detection. (**a**) Stand density, (**b**) Stand location in the road verge, and (**c**) Whether the stand continues outside of the road verge (Yes) or not (No). The letters represent the results from the post-hoc analysis. Comparisons that do not share a letter are significantly different from each other. The error lines represent the standard error.

The results from the analysis of factors influencing probability of detection shown below are based on non-empty stretches only (*n* = 79). In general, the density and position of the lupine stands influenced the probability of detection from the car (Table 3, Table S2). Single plants were harder to detect from the car, but there were no differences in detection probability between single plants and sparse stands. Dense stands had the highest probability of detection (Fig. 5a). Stands that only occurred on one of the slopes (i.e., either side slope or back slope) had lower probability of detection than stands that occurred on both slopes (Fig. 5b). The continuation of the stand outside of the road verge did not influence the detection probability (Fig. 5c). The candidate model had an AICc = 198.12 and the null model had an AICc = 254.82 indicating that the candidate model was better than the null model. There was no evidence of collinearity among explanatory variables (Table 3). Including survey year as an additional random effect did not change parameter estimates, and the associated variance was effectively zero.

**Table 3:** Results from the generalized linear model exploring which characteristics of the lupine stands are important for detection from a moving vehicle. Shown are the Chi-square values (χ^2^), degrees of freedom (df), p-values (p), and variance inflation factors (GVIF^(1/(2*df))^). P-values in bold are significant at the 0.05 level or lower. The estimates, standard errors, z-values, and p-values can be found in Table S1. Marginal R^2^ = 0.44, conditional R^2^ = 0.52.

	χ2	df	*p*	GVIF^(1/(2*df))^
Stand location	16.44	2	**< 0.001**	1.10
Density of the stand	8.24	2	**< 0.01**	1.06
Stand continues outside the road verge	0.00	1	0.99	1.20

**Fig. 6:**
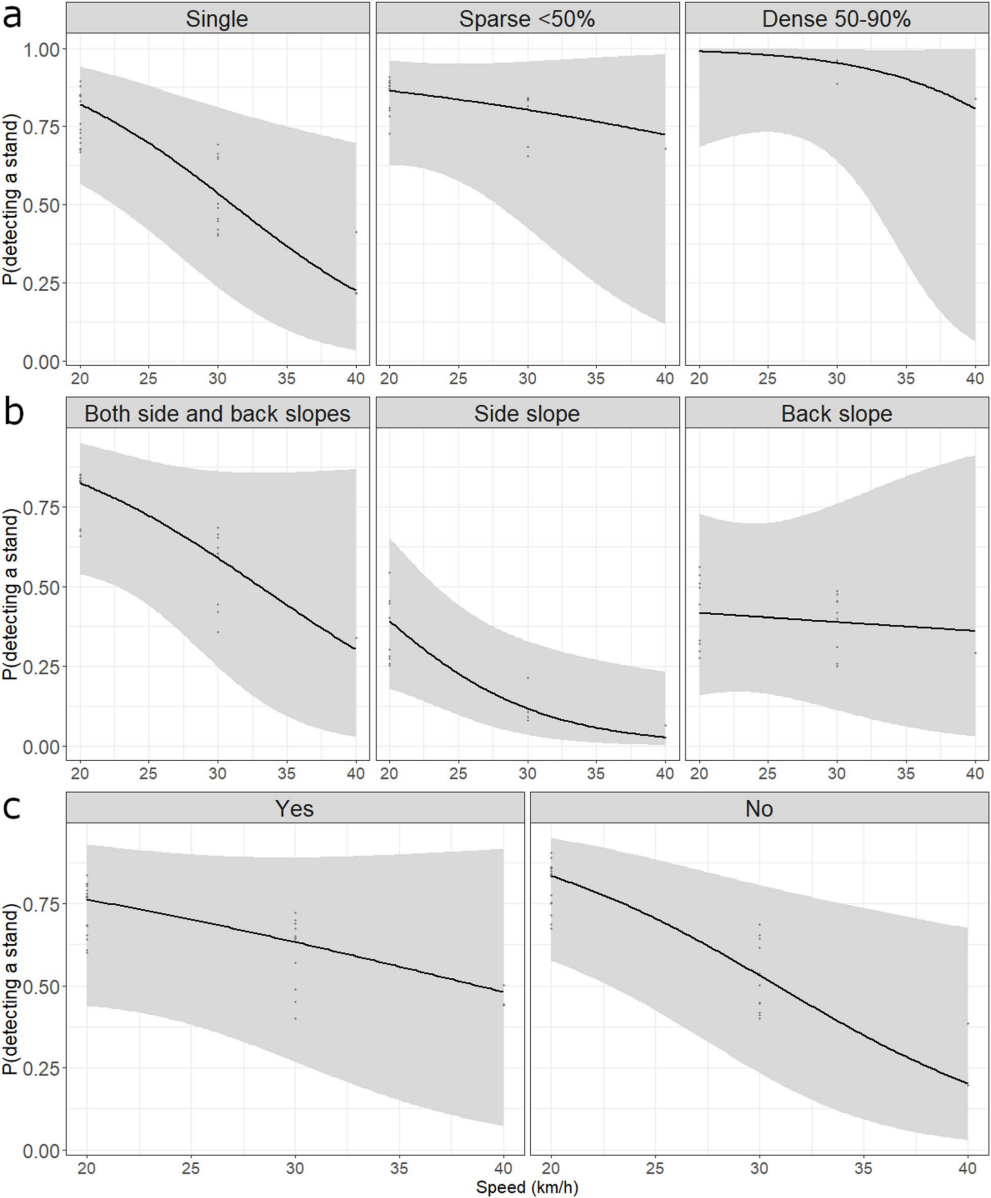
Influence of speed during the survey by car on the probability of detection lupine stands that differ in (**a**) density, (**b**) position in the road verge, and (**c**) whether the stand continues outside of the road verge (Yes) or not (No). The shaded areas represent the 95% confidence intervals. Points represent the individual observations.

The speed of the car influenced the detection of lupine stands during the survey by car. In general, the probability of detecting a stand was higher at lower speeds and steadily decreased with increasing speed (Fig. 6). This pattern was most noticeable for single plants (Fig. 6a) and for stands that were present only on the side slope (Fig. 6b). The probability of detecting dense, i.e. more conspicuous, stands remained high at higher speeds (Fig. 6a). The most parsimonious models were the ones in which the interaction effect was modeled separately for each variable (ΔAICc < 3; Table S3-S6). Therefore, the figures show the results of three separate models in which the interacting effect is added to each explanatory variable separately. Using a subset of the data did not change the results mentioned above, although the p-values increased (Table S7).

## Discussion

Using the survey by foot as a reference method, we showed that the survey by car method is a promising approach for detecting and estimating the abundance of lupines along longer stretches of road verges or in a network of road verges. We found that the detection probability of lupine stands depended on the speed of the car and the density and position of the stand in the road verges but was independent of whether the stand continued also outside of the road verge. Detection success was higher at a larger spatial scale (ca. 85% of 200 m sections correctly classified as containing lupine, compared to ca. 72% of individual stands correctly detected). The survey by car can thus offer road managers a general idea of which road segments require control measures. Given that *L. polyphyllus* is well established in the country, complete eradication at the national level is no longer feasible. In such cases, the survey by car method would be primarily focused on supporting the control and containment of populations to prevent further spread. Identifying areas where eradication is still possible is key to achieve this goal.

As predicted, the density of plants in a stand affected the probability of detecting the stand from the car such that the probability increased with the size of the stand. There was a higher, but not significantly so, probability of detecting sparse stands compared to single plants. (Fig. 5a). However, we suspect that the probability of detection of single plants is even lower than the one we report here, given that stands marked as ‘single plants’ could either be actual single plants or longer line objects with dispersed single plants (which we assume increased their detection probability). Nevertheless, detecting a single plant from the car was less likely in comparison, meaning that the performance of the method will vary depending on the proportion of the total lupine population in an area that consists of single plants. The position of the lupine stands in the road verge also affected their detection probability such that stands that were present on both side and back slopes had a significantly higher detection probability compared to stands that were only present in one of the slopes. Given that the detection probability is assumed to be mostly affected by the size of the individual plants and of the stands, target areas should be surveyed regularly (ideally every year), to allow for single plants or smaller stands to be more easily detected as they grow.

The detection probability increased at lower speeds, with 20 km/h providing the highest probability of detecting lupine stands in the road verge. As the speed of the vehicle increased, the probability of detecting single plants and plants located in the side slope decreased. The reduction in detection probability was most pronounced for single plants, with a drop from ~ 80% probability of detection at 20 km/h to less than 25% at 40 km/h. These results have important implications for planning surveys by car, and survey objectives and availability of resources should guide the choice of driving speed. If the goal is to produce a coarse distribution map of established lupine stands across a broader road network, higher speeds may be acceptable. In contrast, when the objective is early detection of newly established plants, lower speeds are recommended to maximize the probability of detection. Since the cost of eradication of IAP is lowest at an early stage of invasion, there is thus a trade-off between cost of the survey (higher cost at lower speed due to higher time consumption) and cost of eradication (lower cost with earlier detection following lower speed). Nevertheless, our results suggest that detecting single plants is challenging because the probability of detection drops rapidly when the speed exceeds 20 km/h.

Other possible factors influencing detection probability of lupine stands that were not considered in this study are the background vegetation and observer error. For instance, Hauser et al.^12^, found that detection rate of *Pilosella* plants was determined by the interaction between target and background characteristics. In this case, the target and background were the same, that is, lupines and road verges, but variation at a more detailed level might have been of importance. For example, vegetation height surrounding the lupines could have impacted the probability of detection. Furthermore, lupine flowers can adopt a variety of colours such as white, pink, and purple, which could potentially affect their detection probability, and there could be possible differences in detection probability between lupine stands in a road verge next to a forest compared to a road verge next to an agricultural field. Observer error has been repeatedly shown to influence detection probability in plant surveys^32^. We did not explore how the observer influenced detection probability of lupine stands because the identity of the surveyor could not be reliably reconstructed from the database, as the surveyors were working in pairs and shared devices and accounts. Despite this, we believe that blooming individuals of lupines are among the easiest species to identify in the field, and even observers with a low degree of experience in plant surveys should be able to identify them. Consistent with this expectation, we did not record any commission errors (false positives) during the survey by car. This is supported by previous studies, showing that there were no remarkable differences in detection probability between observers when the species are conspicuous and grow tall (e.g.^11^,^37^). When there is no need to differentiate among species, the observer bias should be lower, and detection probability becomes more dependent on the characteristics of the types of stands and the environment they are in. In contrast to detection, estimation of stand density is more subjective^32^, which might have influenced our results and could have been partly controlled for if we had added the observer to the model. However, since we used only three broad density categories, this observer effect is likely limited by the use of broad density categories.

Even though the proposed survey by car method was designed with the lupines in mind, the method could potentially be adapted to surveying other IAP in the road network. Conspicuous species that are easy to identify even for inexperienced observers such as *Solidago canadensis* (Canadian goldenrod), *Impatiens glandulifera* (Himalayan balsam) or *Heracleum mantegazzianum* (giant hogweed) could potentially be surveyed using this method, although with adapted categories of stand density. Due to differing flowering phenology, however, not all of those species can be covered in the same survey. Moreover, IAP with less distinctive morphology may have a higher risk of false positives, which needs to be taken into account when adapting the method to other taxa.

## Conclusions

Road verges have been shown to often have a greater abundance and richness of exotic species and therefore need continuous and cost-effective monitoring of IAP. The suggested method correctly identified 85% of the 200 m sections with lupines, and 72% of the individual lupine stands. In addition, the method correctly identified 100% of the 200 m sections without lupines. If repeated, the method also shows changes in the distribution and abundance of lupines in an area. Based on a distribution map, eradication strategies can be developed. However, given the detection limitations of the survey by car method, eradication measures should be preceded by more detailed surveys and inventories of the stands in and around the chosen areas for eradication in order to find all plants, also early establishments.

The substantial geographical scope allowed by the survey by car method will enable land managers to identify areas to prioritize for eradication of lupine populations. Car-based survey data may also provide a basis for training emerging image-recognition tools for roadside monitoring. The strategy applied to prevent further spread can vary depending on the type of stands and their locations, as well as on the availability of resources. For instance, small and isolated stands (e.g. those consisting of single plants or sparse occurrences) could be prioritized for eradication in order to prevent further establishment into new areas. These stands are both more difficult to detect and may represent early stages of invasion. Since eradication measures may not be 100% effective, and due to some IAP having a seed bank, most eradication measures require continuous monitoring and, if needed, repeated eradication for some years to ensure that the control measure is successful. Alternatively, road stretches with high densities of the invasive could be isolated from the rest of the road network by implementing adaptive management, e.g. cutting before seed-set or by making sure that the machinery used is lupine-free either by cleaning the grass mowers or by using specific grass mowers for lupine-free areas and others for areas with high lupine densities. This strategy requires continuous monitoring of the IAP-free parts of the road network.

## Supplementary Information

Below is the link to the electronic supplementary material.

Supplementary Material 1

## Data Availability

The dataset generated and/or analyzed during the current study are available in FigShare at https://doi.org/10.6084/m9.figshare.31347463.
