# Supplementary material for: Evaluation of a car-based method for detecting the invasive Lupinus polyphyllus Lindl in road infrastructure
Source: Sci Rep. 2026 Jun 25;16:19603. doi: 10.1038/s41598-026-58979-w (PMC13303915; doi:10.1038/s41598-026-58979-w)
Supplement: Supplementary file 1 — Supplementary Material 1 [file 41598_2026_58979_MOESM1_ESM.docx]

**Scientific Reports**

**Supplementary information to the paper Dániel-Ferreira, J. et al.** **Evaluation of a car-based method for detecting the invasive *Lupinus polyphyllus* in road infrastructure**


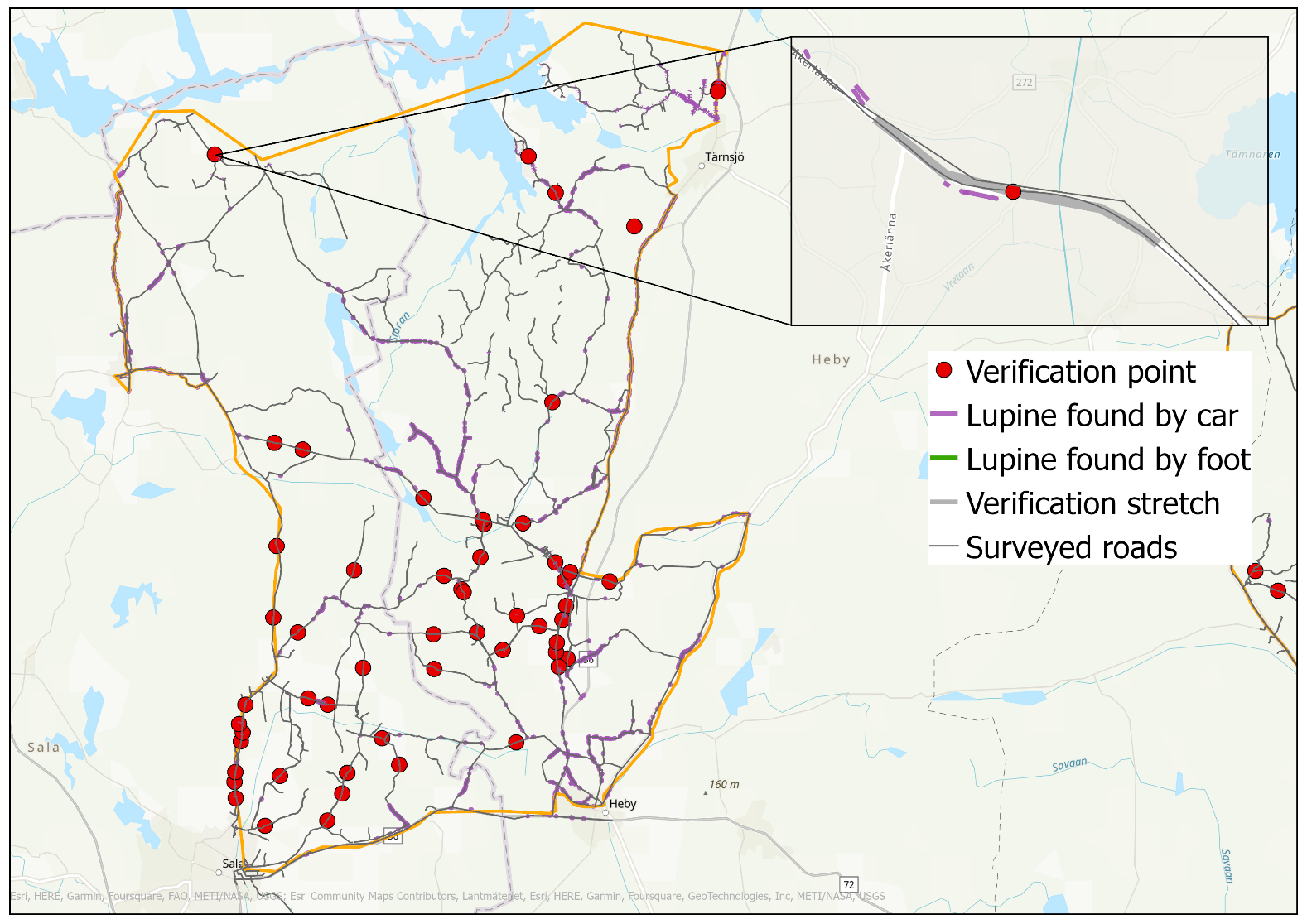


**Figure S1.** Map of the area of Heby depicting the location of the verification points, surveyed roads, and the distribution of lupines within the area. An example of a verification stretch is shown in the top-right corner


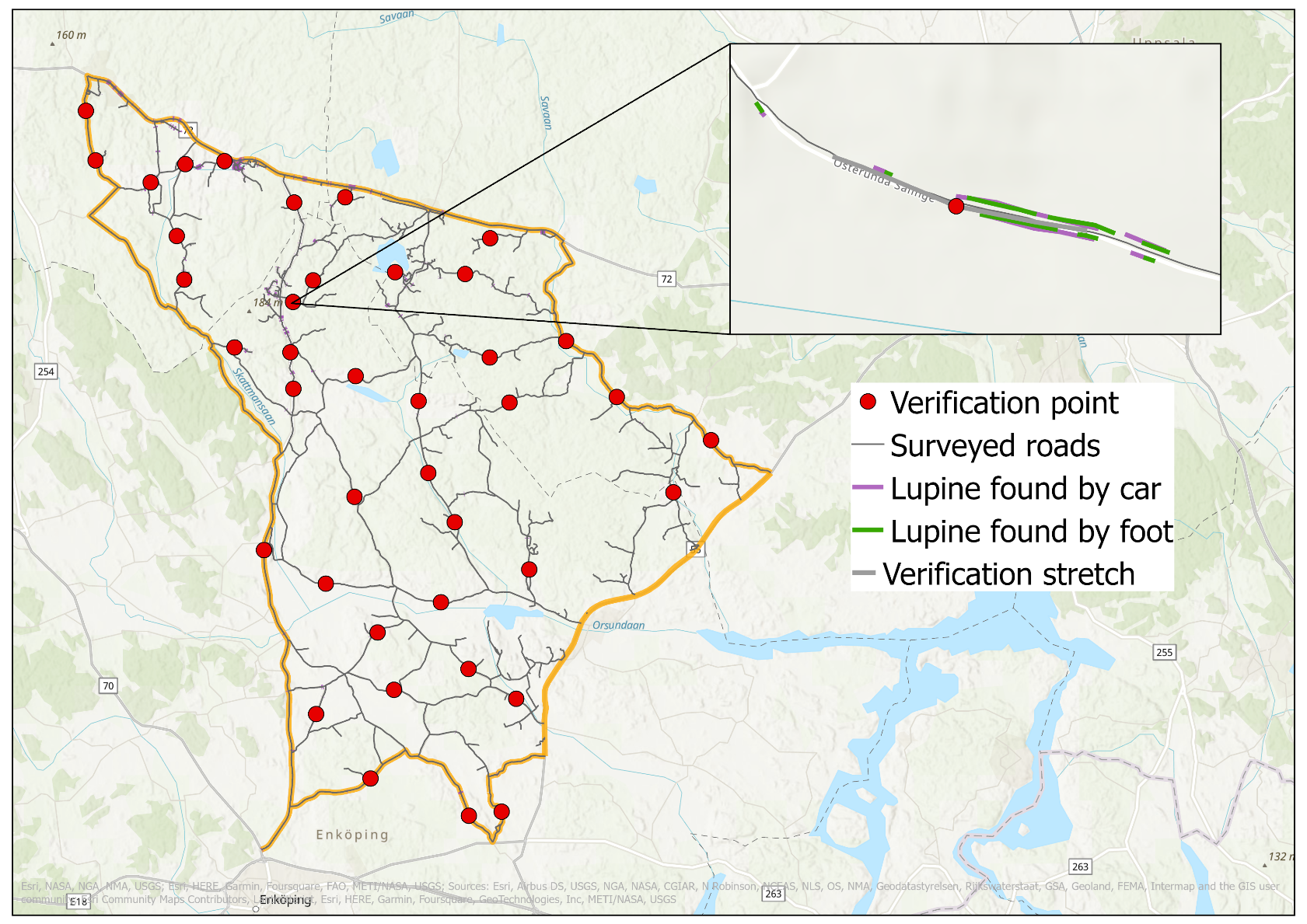


**Figure S2.** Map of the area of Enköping depicting the location of the verification points, surveyed roads, and the distribution of lupines within the area. An example of a verification stretch is shown in the top-right corner


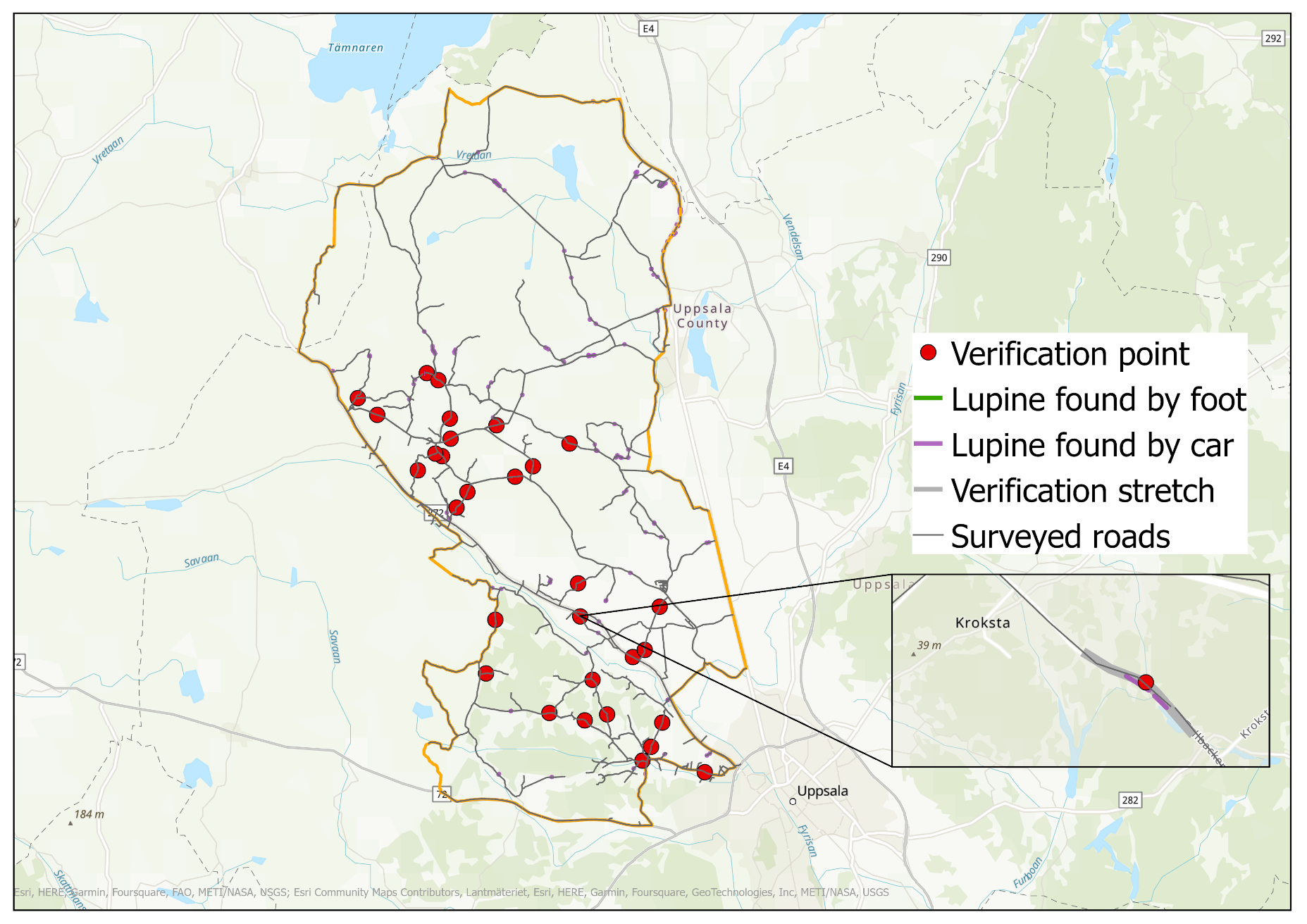


**Figure S3.** Map of the area of Uppsala depicting the location of the verification points, surveyed roads, and the distribution of lupines within the area. An example of a verification stretch is shown in the bottom-right corner


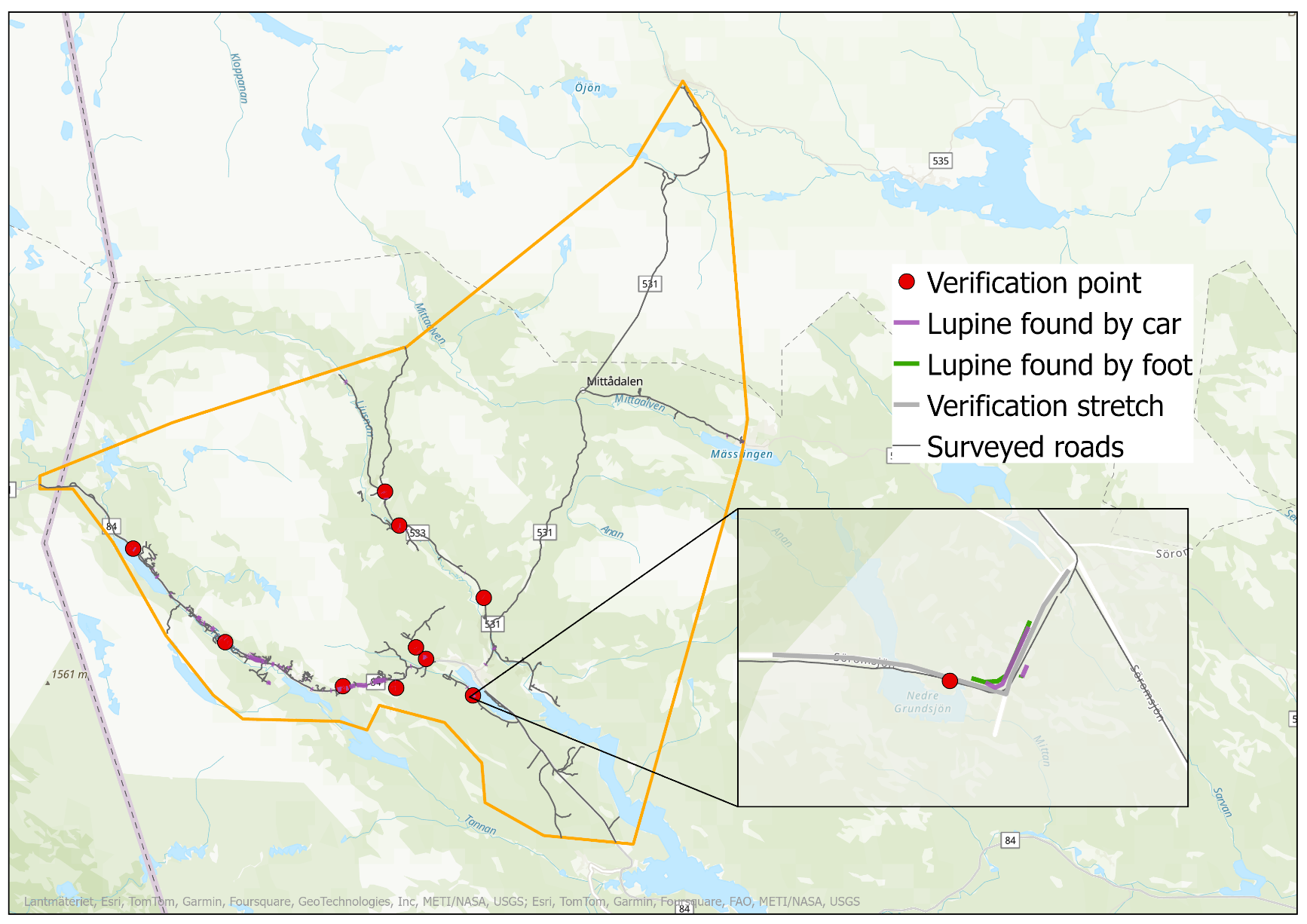


**Figure S4.** Map of the area of Funäsdalen depicting the location of the verification points, surveyed roads, and the distribution of lupines within the area. An example of a verification stretch is shown in the bottom-right corner.


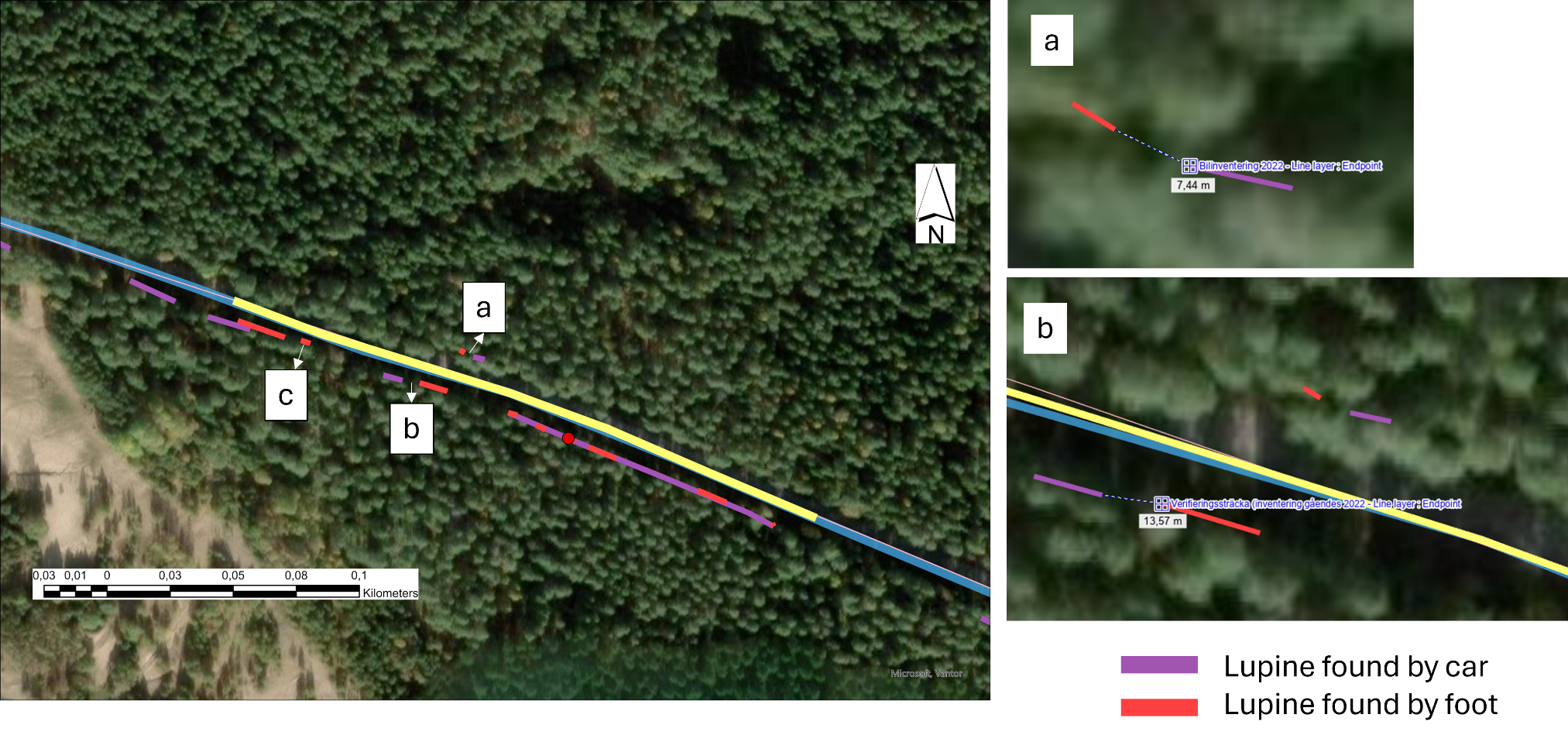


**Fig S5.** Example of the procedure used to assess whether lupine stands identified during the survey by foot were detected during the survey by car. The yellow line indicates the verification stretch. a) Example where the two datasets were matched directly based on spatial proximity (distance between the stands was less than 10 m). b) Example of a match where the distance between objects exceeded 10 m, but correspondence was confirmed based on stand characteristics (stand location and density). c) Example of a stand recorded during the survey by foot that was classified as missed during the survey by car. The rest of the stands were classified as matches.

**Table S1.** Number of observed and missed lupine stands during the survey by foot per study area and year.

| **Area** | **Year of survey** | **Number of observed stands along verification stretches** | **Number of missed stands along verification stretches** |
| --- | --- | --- | --- |
| Heby | 2021 | 21 | 6 |
| Heby | 2022 | 64 | 32 |
| Uppsala | 2021 | 25 | 5 |
| Uppsala | 2022 | 3 | 1 |
| Funäsdalen | 2022 | 16 | 3 |
| Enköping | 2023 | 94 | 15 |

**Table S2.** Results from the generalized linear model exploring which characteristics of the lupine stands are important for detection from a car. Shown are the estimates (e), standard errors (s.e.), z-values (z), and p-values (p). P-values in bold are significant at the 0.05 level or lower.

|  | e | s.e. | z | p |
| --- | --- | --- | --- | --- |
| Intercept | 1.91 | 0.73 |  |  |
| Stand location (Side slope) | -2.15 | 0.54 | -3.96 | **<0.001***** |
| Stand location (Back slope) | -1.80 | 0.65 | -2.77 | **<0.01*** |
| Stand density (Sparse) | 0.88 | 0.44 | 2.02 | **0.04*** |
| Stand density (Dense) | 2.73 | 1.12 | 2.44 | **0.01*** |
| Stand continues outside the road verge (No) | 0.00 | 0.52 | 0.01 | 0.99 |

**Table S3.** AICc values for the candidate models exploring the influence of speed during the survey by car on the probability of detecting lupine stands in the road verge. The top three models were selected, and the results are shown in the tables below and in Figure 6 in the main text.

| Candidate Model | AICc | ΔAICc |
| --- | --- | --- |
| Stand Location + Stand Density + Stand Continues Outside The Road Verge:Speed | **126.93** | **0** |
| Stand Location:Speed + Stand Density + Stand Continues Outside The Road Verge | **128.26** | **1.33** |
| Stand Location + Stand Density:Speed + Stand Continues Outside The Road Verge | **129.24** | **2.31** |
| Stand Location:Speed + Stand Density + Stand Continues Outside The Road Verge:Speed | 130.54 | 3.61 |
| Stand Location + Stand Density:Speed + Stand Continues Outside The Road Verge:Speed | 131.08 | 4.15 |
| Stand Location:Speed + Stand Density:Speed + Stand Continues Outside The Road Verge | 131.61 | 4.68 |
| Stand Location:Speed + Stand Density:Speed + Stand Continues Outside The Road Verge:Speed | 134.06 | 7.13 |
| Stand Location + Stand Density + Stand Continues Outside The Road Verge | 131.98 | 5.05 |
| Null Model | 168.14 | 41.21 |

**Table S4.** Results from the models given in Table S3 (in bold). The model below explored the interaction between speed and the location of the stand in the road verge. Shown are the estimates (e), standard errors (s.e.), z-values (z), and p-values (p). P-values in bold are significant at the 0.05 level or lower.

|  | *e* | *s.e.* | *z* | *p* |
| --- | --- | --- | --- | --- |
| Intercept | 4.46 | 2.16 |  |  |
| Stand Location (Side Slope) | -1.21 | 2.62 | -0.46 | 0.64 |
| Stand Location (Back Slope) | -4.03 | 2.89 | -1.40 | 0.16 |
| Speed | -0.12 | 0.08 | -1.47 | 0.14 |
| Stand Density (Sparse) | 0.61 | 0.60 | 1.02 | 0.31 |
| Stand Density (Dense) | 2.64 | 1.20 | 2.21 | **0.03*** |
| Stand Continues Outside The Road Verge (No) | 0.20 | 0.62 | 0.32 | 0.75 |
| Stand Location (Side Slope):Speed | -0.04 | 0.11 | -0.37 | 0.71 |
| Stand Location (Back Slope):Speed | 0.11 | 0.11 | 0.94 | 0.35 |

**Table S5.** Results from the models given in Table S3 (in bold). The model below explored the interaction between speed and whether the stand continued outside the road verge. Shown are the estimates (e), standard errors (s.e.), z-values (z), and p-values (p). P-values in bold are significant at the 0.05 level or lower.

|  | *e* | *s.e.* | *z* | *p* |
| --- | --- | --- | --- | --- |
| Intercept | 4.92 | 1.68 |  |  |
| Stand Location (Side Slope) | -2.06 | 0.67 | -3.07 | **<0.01*** |
| Stand Location (Back Slope) | -1.39 | 0.73 | -1.91 | 0.06 |
| Stand Density (Sparse) | -1.54 | 2.33 | -0.66 | 0.51 |
| Stand Density (Dense) | 3.46 | 5.00 | 0.69 | 0.49 |
| Speed | -0.14 | 0.06 | -2.43 | 0.01* |
| Stand Continues Outside The Road Verge (No) | 0.21 | 0.63 | 0.34 | 0.73 |
| Stand Density (Sparse):Speed | 0.09 | 0.10 | 0.97 | 0.34 |
| Stand Density (Dense):Speed | -0.02 | 0.17 | -0.12 | 0.91 |

**Table S6.** Results from the models given in table S3 (in bold). The model below explored the interaction between speed and the density of the stand. Shown are the estimates (e), standard errors (s.e.), z-values (z), and p-values (p). P-values in bold are significant at the 0.05 level or lower.

|  | *e* | *s.e.* | *z* | *p* |
| --- | --- | --- | --- | --- |
| Intercept | 3.32 | 1.81 |  |  |
| Stand Location (Side Slope) | -2.14 | 0.69 | -3.11 | **<0.001** |
| Stand Location (Back Slope) | -1.55 | 0.73 | -2.11 | 0.03* |
| Stand Density (Sparse) | 0.52 | 0.60 | 0.86 | 0.39 |
| Stand Density (Dense) | 2.50 | 1.17 | 2.13 | 0.03* |
| Stand Continues Outside The Road Verge (No) | 2.20 | 2.24 | 0.98 | 0.33 |
| Speed | -0.06 | 0.07 | -0.95 | 0.34 |
| Stand Continues Outside The Road Verge (No):Speed | -0.09 | 0.09 | -098 | 0.33 |

**Table S7.** Results from the model using the same explanatory variables as the model using the complete data set (AICc = 131.98). Shown are the estimates (e), standard errors (s.e.), z-values (z), and p-values (p). P-values in bold are significant at the 0.05 level or lower.

|  | e | s.e. | z | p |
| --- | --- | --- | --- | --- |
| Intercept | 1.38 | 0.82 |  |  |
| Stand location (Side slope) | -2.32 | 0.64 | -3.62 | **<0.001***** |
| Stand location (Back slope) | -1.31 | 0.70 | -1.88 | 0.06 |
| Stand density (Sparse) | 0.97 | 0.56 | 1.73 | 0.08 |
| Stand density (Dense) | 2.78 | 1.14 | 2.44 | **0.01*** |
| Stand continues outside the road verge (No) | 0.24 | 0.56 | 0.40 | 0.69 |
